# Embedding health and wellbeing opportunities for people experiencing homelessness in a wider support system

**DOI:** 10.1177/17579139231157527

**Published:** 2023-05-26

**Authors:** M Paisi, L Withers, J Erwin, J Horrell, R Witton, J Shawe, R Byng

**Affiliations:** Peninsula Dental School, University of Plymouth, Plymouth, UK; School of Nursing and Midwifery, University of Plymouth, Plymouth, UK; Research and Community Partner, Plymouth, UK; Peninsula Dental School, University of Plymouth, Plymouth, UK; Peninsula Dental School, University of Plymouth, Plymouth, UK; Peninsula Dental School, University of Plymouth, Plymouth, UK; School of Nursing and Midwifery, University of Plymouth, Plymouth, UK; Peninsula Medical School, University of Plymouth, Plymouth, UK

## Introduction

Homelessness impacts negatively on health, wellbeing and life expectancy. People experiencing homelessness are likely to suffer physical and mental health problems, be heavy users of emergency services, and die 30 years earlier than the general population.^1–3^ Their severe and multiple disadvantages raise personal and institutional barriers to using health, social and housing services.^4^

In late 2021, a grass-roots initiative in Plymouth began offering a Saturday morning drop-in service for rough sleepers and those in emergency accommodation. The service responded to the bleakness, loneliness and lack of support imposed by Monday-Friday service patterns. This effort was strengthened in February 2022 by a six month Plymouth University grant that enabled partners with extensive experience in the homelessness sector to collaborate with health and wellbeing practitioners in a project aiming to:

- Meet basic human needs for nutrition, personal hygiene and connectedness.
- Offer weekly engagement opportunities with activities supporting health/wellbeing, recovery and personal development.
- Provide data to evidence client needs and improve engagement with health/wellbeing opportunities.

## Methods

The Plymouth Alliance^5^ coordinates a partnership of local homelessness and health organisations supporting people with complex needs. The project was run by staff from two Alliance member charities, Plymouth Access to Housing (Path) and Shekinah, and volunteers from Plymouth Soup Run. Other Alliance staff joined the project as volunteers. All people accessing the service (‘clients’) were offered a cooked breakfast, a shower, clean clothing and a takeaway lunch. Rough sleepers were offered sleeping bags. Weekly engagement opportunities with at least one healthcare provider addressed: oral health, footcare, bloodborne virus (BBV) testing, eyecare, general nursing, mental health, and smoking cessation. Recreational activities included art sessions and board games.

Attendance data and client needs were recorded each session. A researcher and a peer advocate evaluated sessions from client, staff, volunteer and manager perspectives via interviews and a focus group. Visiting practitioners’ experiences were recorded via questionnaires. In addition, client feedback was obtained ad hoc during sessions. The project leads, functioning as embedded volunteer researchers,^6^ developed the evaluation framework and recorded personal reflective notes. This work was part of service monitoring and improvement and included non-identifiable information. Hence ethical approval was not required and individuals provided a verbal consent.

**Figure:**
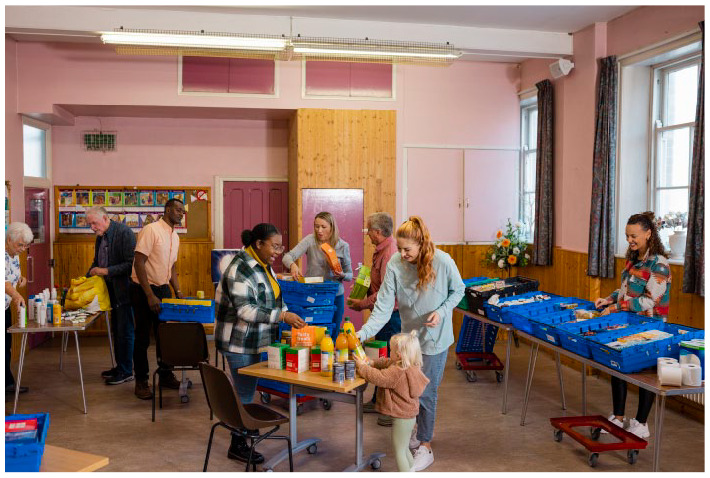


## Findings

### Attendance

In six months,174 clients accessed the sessions (25 (14%) women; 149 (86%) men). There were 500 attendances, averaging 19 per week. Most clients were rough sleeping (59%) or in emergency/supported accommodation (37%). The project’s reach grew from 60% of Plymouth’s evidenced rough sleepers in February to over 80% by July 2022.

### Service evaluation

The Saturday morning sessions met the need for somewhere for rough sleepers and those in emergency accommodation to go at weekends in a safe, quiet environment supporting practical, health and social needs. The sessions facilitated focused work rather than the ‘firefighting’ commonly experienced by support workers. Careful management of admissions and swift defusing of tension maintained the sense of a controlled, stress-free environment.

Clients received help with housing from volunteers working in the field. These interactions gave the volunteers a greater understanding of clients’ individual situations, and the ability to make more nuanced decisions than those based on stark records. Mutual understanding was developed, tempering client hostility towards those ‘in authority’. Accommodation was secured ‘out-of-hours’ for particularly vulnerable rough sleepers.

Staff and volunteers appreciated the links built between organisations that supported collaborative working with wide benefits for clients. Getting to know clients as individuals was valued and clients appreciated being able to have ‘normal’ conversations, where they could share thoughts, and not feel like a ‘case to be solved’ or ‘a number in a system’.

The need was recognised for a flexible approach for people who may struggle with making and attending appointments. The benefits of interdisciplinary working and trauma-informed approaches were highlighted, along with awareness of the needs of people at critical transition points such as hospital discharge or release from custody.

### Engagement with healthcare and art sessions

Clients were generally keen to engage with healthcare professionals whose presence within a familiar service supported the development of trusting relationships. Healthcare assessment and treatment have promoted prevention and facilitated referral to other healthcare providers, plus immediate treatment of conditions that would otherwise escalate. Oral health educators proactively interacted with the majority of clients present, whereas some other services, for example, podiatry or BBV testing, reached 30% to 40% of those present through self-selection and targeting. Fewer clients (ca. 20%) engaged with mental health peer mentors, but this engagement yielded some very effective outcomes over time.

Not all healthcare needs could be met within the Saturday sessions. While dental professionals could deliver oral healthcare messages, acute intervention was only possible by signposting to an emergency dentist. Podiatrists made referrals to the outreach general practitioner (GP) service and the local emergency department (ED), and mental health peer mentors connected clients with support groups.

The focus of the art sessions included making Easter decorations, mindful colouring, printing and expressive painting. Engagement varied from one or two clients to 30% of all present, some engaging briefly and others immersing themselves in a welcome distraction from everyday concerns. These sessions stimulated rich conversations around life experiences, worries and hopes.

## Conclusions

The project was successful in meeting its aims, due not least to the presence of embedded volunteer researchers. This created trust and enhanced interaction across the network of stakeholders, including clients. It facilitated effective evaluation and learning for practice improvement and capacity building.

The Saturday sessions are continuing despite the termination of grant funding. These sessions seek to offer ‘normalising’ experiences: casual conversation, rare opportunities to make choices about food and clothing offered, the possibility of joining creative activities, and the chance to deal with health issues before they become emergencies. All of these elements can get squeezed out of a life impacted by homelessness.

Food brings people together, creating an environment where wider support can be offered. By definition, housing advice is emphasised as a priority need for the client group, but health concerns merit attention to support transition from homelessness to a more stable life. It is clear that taking services to people works.

Client circumstances can change rapidly, compounded by physical and mental health constraints of the lived experience of homelessness. Trauma and shame surfaced as issues for many, leading to low health expectations. Hopefully, the supportive environment offered clients dignity and encouragement to seek help.

It is valid to ask whether the Saturday service is supporting clients’ progress or enabling the status quo. In response, it is felt that the service does not incentivise rough sleeping. On the contrary, it is a vital avenue for contact with people suffering severe and multiple disadvantages who often fall outside the reach of regular services.

However, it is also considered that the Saturday service needs to be part of a bigger picture of comprehensive, joined-up and personalised support for clients, giving them the prospect of a different future. Help with physical health, mental health and addictions, plus opportunities for meaningful occupation would all be a part of that offer.
